# Seasonal Allergies and Psychiatric Disorders in the United States

**DOI:** 10.3390/ijerph15091965

**Published:** 2018-09-08

**Authors:** Hans Oh, Ai Koyanagi, Jordan E. DeVylder, Andrew Stickley

**Affiliations:** 1 Suzanne Dworak-Peck School of Social Work, University of Southern California, 669 W. 34 th St., Los Angeles, CA 90089-0411, USA; 2 Parc Sanitari Sant Joan de Déu, Universitat de Barcelona, Fundació Sant Joan de Deu, Dr. Antoni Pujadas, 42, Sant Boi de Llobregat, 08830 Barcelona, Spain; a.koyanagi@pssjd.org; 3 Instituto de Salud Carlos III, Centro de Investigación Biomédica en Red de Salud Mental, CIBERSAM, Monforte de Lemos 3-5 Pabellón 11, 28029 Madrid, Spain; 4 Graduate School of Social Service, Fordham University, 113 W 60th Street, New York, NY 10023, USA; jdevylder@fordham.edu; 5 The Stockholm Center for Health and Social Change (SCOHOST), Södertörn University, 141 89 Huddinge, Sweden; amstick66@gmail.com; 6 Department of Preventive Intervention for Psychiatric Disorders, National Institute of Mental Health, National Center of Neurology and Psychiatry, 4-1-1 Ogawahigashicho, Kodaira, Tokyo 1878553, Japan

**Keywords:** Allergies, allergic rhinitis, psychiatric disorders, Latinos, Asians, African Americans

## Abstract

Seasonal allergies have been associated with mental health problems, though the evidence is still emergent, particularly in the United States. We analyzed data from the National Comorbidity Survey Replication and the National Latino and Asian American Survey (years 2001–2003). Multivariable logistic regression models were used to examine the relations between lifetime allergies and lifetime psychiatric disorders (each disorder in a separate model), adjusting for socio-demographic variables (including region of residence) and tobacco use. Analyses were also stratified to test for effect modification by race and sex. A history of seasonal allergies was associated with greater odds of mood disorders, anxiety disorders, and eating disorders, but not alcohol or substance use disorders, after adjusting for socio-demographic characteristics and tobacco use. The associations between seasonal allergies and mood disorders, substance use disorders, and alcohol use disorders were particularly strong for Latino Americans. The association between seasonal allergies and eating disorders was stronger for men than women. Seasonal allergies are a risk factor for psychiatric disorders. Individuals complaining of seasonal allergies should be screened for early signs of mental health problems and referred to specialized services accordingly.

## 1. Introduction

Across the globe, evidence indicates that environments are transforming rapidly, spurring researchers to increasingly examine how changes in the ecosystem impact human health. Warmer climates may be lengthening the ragweed pollen season in North America [1]; pollution created by humans may also be increasing carbon dioxide levels in the atmosphere, contributing to higher pollen counts in Europe [2]; and higher levels of pollution may be exacerbating the effects of airborne allergens in Asia [3]. These conditions can have an impact on allergic and respiratory diseases [4], which some studies suggest have increased in the past decades in various parts of the world [5,6,7]. It is estimated that approximately 8.2% of the population suffer from hay fever in the United States, which in some instances can pose a serious burden [8], though seldom a life-threatening one. Interestingly, recent evidence suggests that the burden of seasonal allergies may be linked to significant mental health problems [9].

Seasonal allergies (such as hay fever) occur when the immune system overreacts to allergens, resulting in sneezing, itchy and watery eyes, runny nose, and other symptoms. The immune system’s response to pollen and other allergens can trigger the release of proinflammatory cytokines that affect monoaminergic neurotransmission [10,11], potentially contributing to the pathogenesis of mental health problems [12,13,14,15,16,17,18]. Indeed, studies have documented the relations between inflammatory/immunological factors and a wide range of mental health problems [11,12,19,20] as well as substance use disorder [21]. Moreover, allergies may result in lower perceived health-related quality of life [22].

Research on the relation between seasonal allergies and mental health is still in its early stages. Studies have found that allergic rhinitis is associated with mood disorders [9,23,24,25,26], suicidal ideation [27], and completed suicide [28], with some evidence of an association with anxiety disorders [23,29], but not substance use disorders [29]. To our knowledge, as yet, very few studies have examined the relation between seasonal allergies and alcohol or substance use disorders or eating disorders, even though these conditions are also linked to inflammation [21,30].

Studies have offered mixed evidence regarding the role of allergies in mental health, depending on the specific outcome and population being examined. The United States is a large and culturally diverse country, with variations in allergic diseases and mental health observable across racial and ethnic categories [31,32]. While genetic factors have been posited to explain racial differences, one study showed that genetic ancestry is not significantly associated with allergic sensitization after adjusting for location of residence [33], stressing the importance of environmental factors, which are largely socially determined and are patterned according to race and sex. There is some evidence that the association between seasonal allergies and mental health outcomes may differ between different ethnic groups [27]. Similarly, associations between allergies and mental health outcomes may also vary across sex [28,34,35]. Modification of these associations by race or sex may reflect differential exposures to allergens based on location (e.g., climate, flora, neighborhood/housing conditions), differential exposures to stressors (which can lead to immune alterations), or even differences in treatment adherence/outcomes [36,37,38,39].

In the current study, we analyzed data from the National Comorbidity Survey Replication and the National Latino and Asian American Survey, which taken together provide a robust racially and ethnically diverse sample of the United States general population. We examined the association between seasonal allergies and major psychiatric disorders. In a set of exploratory analyses, we test for effect modification by race and by sex, which can lay the groundwork for more targeted studies of at-risk populations.

## 2. Methods

### 2.1. Sample

We analyzed data from the Collaborative Psychiatric Surveys (CPES), which comprise two household surveys: (1) the National Comorbidity Survey Replication (NCS-R); [40], (2) the National Latino and Asian American Study (NLAAS); [41]. These surveys were conducted between 2001 and 2003, and used a common core instrument, and similar multi-stage probability sampling strategies to achieve nationally representative samples of adults in the general population of the United States. The NCS-R is a nationally representative survey of 9282 individuals (predominantly White, reflecting the general population of the United States). The NLAAS is a nationally representative sample of Latino Americans (*n* = 2554) and Asian Americans (*n* = 2095). Sampling methodology of the CPES is described in detail elsewhere [42,43]. Survey weights were available to allow for the datasets to be merged. 

### 2.2. Measures

Lifetime seasonal allergies (Main Predictor). In the NCS-R and NLAAS, seasonal allergies were measured using a single self-reported dichotomous item: Have you ever had seasonal allergies (hay fever)? 

Lifetime Psychiatric Disorders (Main Outcomes). Lifetime psychiatric disorders were based on the Word Mental Health—Composite International Diagnostic Interview [44], a fully structured lay interview to screen for diagnoses according to DSM-IV criteria. Lifetime psychiatric disorders included: mood disorder (dysthymia, depressive episode, major depressive disorder), anxiety disorder (agoraphobia with and without panic disorder, generalized anxiety disorder, panic attacks, panic disorder, post-traumatic stress, social phobia), substance use disorder (drug abuse and dependence), alcohol use disorder (alcohol abuse and dependence), and eating disorders (anorexia, binge eating, bulimia).

Covariates. All models were adjusted for age (18–29, 30–44, 45–59, ≥60), and sex (male, female), education (less than high school, high school graduate, some college, college graduate/beyond college), and race (White, Black, Latino, Asian, Other). Models were also adjusted for income using the poverty-income ratio, whereby a ratio of 1 equals the poverty line. Thus, a ratio of 0–1 was considered poor, while values greater than 1 but less than 2 were considered near-poor (e.g., a ratio of 1.5 signifies an income that is 1.5 times the federal poverty line). Values above 2 were considered non-poor. The types and levels of allergens that one is exposed to may depend on geography and climate. For instance, wind and rainfall can influence pollen counts, and molds can grow more quickly with humidity. As such, we controlled for region of residence in the country (West, Northeast, South, and Midwest). Further, we also control for tobacco use, which has been linked to upper respiratory conditions and has been known to increase allergic rhinitis and the allergic response [45,46], and can lead to stress and neurogenic inflammation that may be connected to mental health problems [11,47,48]. Tobacco use was defined using a dichotomous variable indicating current/former smoker vs. never.

### 2.3. Analyses

The NCS-R and NLAAS datasets were merged and analyzed together. Standard errors were estimated through design-based analyses that used the Taylor series linearization method to account for the complex multistage clustered design, with US metropolitan statistical areas or counties as the primary sampling units. Sampling weights were used for all statistical analyses to account for individual-level sampling factors (i.e., non-response and unequal probabilities of selection). Significance was set at *a* = 0.05, two-tailed. All analyses were performed using STATA SE 13 (StataCorp LP, College Station, TX, USA). Multivariable logistic regression models were used to examine the relations between lifetime allergies and lifetime psychiatric disorders (each psychiatric disorder in a separate model), adjusting for socio-demographic variables (including region of residence) and tobacco use. Analyses were then stratified by sex and by race.

## 3. Results

Descriptive statistics for the analytic sample are presented in Table 1. Just over one-third i.e., 36.58% of the entire weighted sample reported lifetime allergies. Similarly, 36.49% of the weighted sample reported at least one type of anxiety disorder at some point in time, which was almost double the lifetime prevalence of mood disorders (19.26%), while substance use disorders and eating disorders were relatively uncommon.

**Figure 1:**
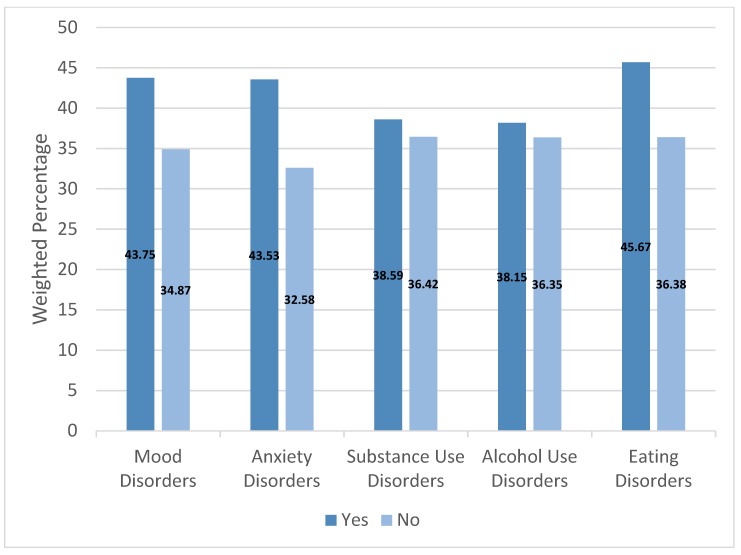
Prevalence of lifetime allergies by lifetime psychiatric disorder (United States, 2001–2003).

The percentage of individuals with lifetime allergies was significantly greater among people with lifetime mood, anxiety, and eating disorders, but not among people with substance or alcohol use disorders (Figure 1; Table S1).

### 3.1. Main Effects

When examining the entire sample, having a lifetime allergy was significantly associated with 28% greater odds of reporting mood disorders, 43% greater odds of reporting anxiety disorders, and 38% greater odds of reporting eating disorders, when compared with individuals who did not have allergies, adjusting for demographic characteristics and a history of smoking. Allergies were not significantly associated with substance use or alcohol use disorders (Table 2).

### 3.2. Effect Modification

When stratifying by race, the magnitude of the effect was stronger among Latino Americans for mood disorders, substance use disorders, and alcohol use disorders, when compared with other racial groups (Table 3). When stratifying by sex, allergies were more strongly associated with eating disorders among men (Table 3).

## 4. Discussion

This study shows that a history of seasonal allergies was associated with significantly higher odds of reporting lifetime mood disorders, anxiety disorders, and eating disorders. The strength of this study is that it used large ethnically diverse probability samples of the general U.S. population, and validated measures of DSM-IV psychiatric disorders. Consistent with previous studies, our main analysis showed that seasonal allergies were associated with mood disorders. While findings from the extant literature have been mixed about whether seasonal allergies are associated with anxiety disorders, the results of this study suggest that a statistically significant association does exist in the U.S. population. Consistent with one prior study [29], our main analysis showed no significant association between seasonal allergies and alcohol or substance use disorders. To our knowledge, this study is one of the few studies that has examined seasonal allergies and eating disorders [49], and potentially the only study to have done so in the United States. We found that seasonal allergies were associated with a greater risk for eating disorders.

As stated earlier, seasonal allergies may be related to inflammation that putatively influence monoaminergic systems that are implicated in psychiatric disorders. However, the association is largely confounded by psychological distress, which is central to the etiology of psychiatric disorders but can also give rise to allergies. Neuroendocrine hormones released in response to stressful events (e.g., traumas, chronic stressors) can suppress the immune system, alter immune function (i.e., dysregulating the type 2 cytokine response), and change the course of immune-based diseases [50]. One of our novel findings is that seasonal allergies were significantly associated with eating disorders. It is possible that individuals might reduce food intake as a reaction to the pro-inflammatory cytokines that are released in their bodies in response to stress, infections, or injuries [51]. Still, it is unknown why seasonal allergies would be associated with an increased risk for some psychiatric disorders but not others.

Conceivably, certain racial groups may have disproportionate exposures to specific allergens that cause increased sensitization to the allergen [32,33]. Studies have shown that allergic diseases are distributed more heavily among those with low socioeconomic status, possibly reflecting greater exposure to certain types of allergens [52]. One study found that poor socioeconomic conditions were associated with high levels of cockroach allergen, but lower levels of dust mite allergen, while higher socioeconomic conditions were associated with high levels of dust mite allergens, and suburban environments were associated with exposure to pollen-related aeroallergens [53]. Socioeconomic status in the United States is inextricably tied to race/ethnicity, as Black and Latino Americans are more likely than their White counterparts to reside in low-income neighborhoods [54,55]. In our analyses, we controlled for both income and education, and still found effect modification by race, implicating other determinants of immunologic susceptibility beyond socioeconomic status. Contrary to previous research [32,33,34,52], we did not find that the relations between allergies and psychiatric disorders were notably stronger among Black or Asian Americans. However, we found that for Latinos, seasonal allergies did in fact increase the risk for alcohol and substance use disorders, and that the relations between seasonal allergies and mood disorders were stronger when compared with Whites, which to our knowledge, has not been seen in prior studies.

In the absence of literature on effect modification across racial groups, we can only offer speculations about our findings. It is possible that racial and ethnic minority groups may reside in communities where they are exposed to specific allergens that result in sensitization and more severe inflammation. That is, one’s geographic location determines the type and amount of allergens that one is exposed to, and geographic location is often a function of racial patterning (e.g., segregation, migration, containment, ethnic enclaves) in the United States. For example, when compared with White children, African American and Mexican American children have higher odds of having cockroach or dust mite sensitivity [56], which may be attributable to residing in low income, densely populated areas, in crowded multi-family homes, where cockroach allergens are more prevalent. However, greater exposure and subsequent sensitization is an imperfect narrative that fails to adequately explain why an effect modification was observed for Latinos but not Black Americans in the current study.

Speculatively, much of the modulation of immunity may be stress-induced, and so future studies may consider the unique stressors that Latinos face in the United States. Latinos also may be less likely to engage in health care (due to language barriers), and may have greater difficulties receiving timely vaccinations, antibiotics, or other medications to manage allergic diseases. Thus, Latinos may be more likely to remain undiagnosed (underutilizing allergy testing) or under-report allergy symptoms. There may also be cultural differences in terms of diet, hygiene, and lifestyle practices (e.g., using a clothesline vs. machine dryer to do laundry). However, these are merely speculations that should be tested in future research. The moderating effect for Latinos was not evident for anxiety disorders, adding an additional layer of complexity that should inspire future studies to explore how differential exposures to specific allergens are related to specific psychiatric disorders across racial groups.

Overall, we did not find effect modification by sex for most psychiatric disorders with the exception of eating disorders. Some research has indicated that there is a higher prevalence of rhinitis and hay fever among females [34], and have observed a greater negative effect of allergy/allergens on mental health among women [28,35]. We did not find this to be true in our study, but we did find that the association between allergies and eating disorders was stronger among men than it was for women. Eating disorders were rare in this sample (2.16%), and were more prevalent among women than men, which is generally true in the United States [57]. Men in some instances may even report a higher prevalence of overeating when compared with women in some (but not all) studies [58,59]. An important task for future research will be to further elucidate the association between allergic disease and eating disorders and the factors that might underlie any sex differences in this relation.

Our findings should be interpreted keeping in mind several potential limitations. First, the data were cross-sectional, making it impossible to establish the temporal order of events or make any causal inferences. Second, the allergy variable was measured using a single self-reported dichotomous item, which did not provide details of the specific type of allergy or the course and severity of the symptoms associated with it. This absence of information was further compounded by the fact that we lacked information on treatment behavior, which is an important omission because as we only examined the lifetime occurrence of seasonal allergies, it is possible that many individuals might have successfully managed their allergies with medications, resulting in biases. Lastly, it was not possible to explore the putative mechanisms that link allergies to mental health problems.

## 5. Conclusions

This study shows that a history of seasonal allergies was associated with significantly higher odds for lifetime mood disorders, anxiety disorders, and eating disorders, but not alcohol or substance use disorders. After testing for effect modification, however, seasonal allergies were strongly associated with mood, alcohol, and substance use disorders among Latinos only (when compared with Whites). Seasonal allergies were associated with eating disorders more strongly among men than among women. A major implication of these findings is the need for early integrated care, such that children and youth complaining of allergy symptoms in family care settings can be quickly screened for early signs of mental health problems, and can be referred accordingly to specialized preventive mental health treatment. Culturally tailored outreach efforts should target Latino Americans in the community in light of major treatment gaps [60]. It remains to be seen whether or not over-the-counter anti-histamines would actually help improve mental health, as one study showed that using anticholinergics could be linked to dementia [61]. And so an area for future research may be testing the effect of nasal steroids or long-term immunotherapy in curbing the inflammatory cascade, which may in some way prevent or alleviate mental health problems [62]. Finally, future studies should continue to examine the intersections of race/ethnicity, socioeconomic status, and sex, to assess how social forces shape the associations between seasonal allergies and immunologic diseases.

## Figures and Tables

**Table 1:** Analytic sample characteristics of the NCS-R and NLAAS (United States, 2001–2003) (*N* = 10,334).

Main Predictor	*N*	Weighted % (Standard Error)
Allergies (lifetime)	3522	36.58 (1.15)
Main Outcomes		
Psychiatric Disorders (lifetime)		
Mood Disorders	2501	19.26 (0.67)
Anxiety Disorders	4096	36.49 (1.18)
Substance Use Disorders	831	7.52 (0.36)
Alcohol Use Disorders	1333	12.61 (0.50)
Eating Disorders	314	2.16 (0.20)
Covariates		
Sex		
Male	4518	47.33 (0.84)
Female	5845	52.67 (0.84)
Race		
White	4191	70.72 (1.64)
Asian	2178	4.31 (0.39)
Latino	3083	11.71 (0.90)
Black	722	11.19 (0.95)
Other	189	2.07 (0.25)
Age		
18–29	2625	23.31 (1.01)
30–44	3537	29.25 (0.83)
45–59	2608	26.21 (0.94)
60+	1593	21.23 (0.97)
Income		
Poor	968	7.78 (0.52)
Near poor	3122	29.43 (1.02)
Non-poor	6244	62.79 (1.14)
Education		
Less than high school	2161	17.98 (0.77)
High school graduate	2718	31.18 (1.05)
Some college	2818	27.56 (0.72)
College graduate and beyond	2666	23.28 (0.96)
Region		
West	3638	23.73 (1.88)
Northeast	1848	19.03 (2.82)
South	3047	34.22 (1.85)
Midwest	1830	23.02 (1.74)
Tobacco use *	4685	48.73 (0.97)

* Tobacco use refers to individuals who were current or former smokers. NLAAS: National Latino and Asian American Study; NCS-R: National Comorbidity Survey Replication.

**Table 2:** Multivariable logistic regression models depicting the associations between lifetime seasonal allergies and lifetime psychiatric disorders (*N* = 10,334).

Psychiatric Disorders	Unadjusted OR (95% CI)	AOR (95% CI)
Mood Disorders	1.45 (1.26–1.67) ***	1.28 (1.10–1.49) **
Anxiety Disorders	1.60 (1.42–1.78) ***	1.43 (1.27–1.63) ***
Substance Use Disorders	1.10 (0.85–1.42)	1.19 (0.89–1.59)
Alcohol Use Disorders	1.08 (0.89–1.32)	1.20 (0.99–1.46)
Eating Disorders	1.47 (1.12–1.93) **	1.38 (1.03–1.84) *

* *p* < 0.05, ** *p* < 0.01, *** *p* < 0.001. Each psychiatric disorder was examined in separate adjusted models. All models were adjusted for age (18–29, 30–44, 45–59, 60–100), sex (ref: male), race (Black, Asian, Latino, Other, White), income (poor, near poor, non-poor), education (less than high school, high school graduate, some college, college graduate and beyond), region of the country, (West, Northeast, Midwest, South), and tobacco use (former or current smoker vs. never).

**Table 3:** Multivariable logistic regression models depicting the associations between lifetime seasonal allergies and lifetime psychiatric disorders, with effect modification by sex and race.

Psychiatric Disorders	Stratified by Sex		Stratified by Race				
	Men	Women	Whites	Asian	Latino	Black	Other
	*N* = 4503	*N* = 5831	*N* = 4175	*N* = 2178	*N* = 3079	*N* = 717	*N* = 185
Mood Disorders	1.33 (1.11–1.60) **	1.26 (1.02–1.57) *	1.18 (1.00–1.39) *	1.27 (0.85–1.90)	1.74 (1.31–2.30) ***	1.54 (0.92–2.58)	1.34 (0.78–2.30)
Anxiety Disorders	1.44 (1.18–1.76) **	1.43 (1.21–1.71) ***	1.42 (1.20–1.67) ***	1.44 (1.11–1.87) **	1.62 (1.34–1.96) ***	1.42 (0.91–2.22)	1.99 (0.77–5.14)
Substance Use Disorders	1.32 (0.94–1.85)	0.98 (0.68–1.41)	1.12 (0.77–1.64)	0.66 (0.27–1.63)	2.11 (1.40–3.18) ***	0.96 (0.44–2.09)	1.00 (0.16–6.06)
Alcohol Use Disorders	1.33 (1.07–1.65) *	0.97 (0.68–1.38)	1.11 (0.88–1.40)	1.23 (0.51–2.96)	1.84 (1.29–2.63) **	1.69 (0.86–3.35)	1.03 (0.52–2.05)
Eating Disorders	2.74 (1.70–4.41) ***	1.03 (0.70–1.51)	1.45 (0.98–2.14)	1.31 (0.69–2.48)	1.48 (0.88–2.48)	0.76 (0.17–3.36)	1.63 (0.17–15.99)

Odds Ratios (95% Confidence Intervals). * *p* < 0.05, ** *p* < 0.01, *** *p* < 0.001. Each psychiatric disorder was examined in separate adjusted models. All models were adjusted for age (18–29, 30–44, 45–59, 60–100), income (poor, near poor, non-poor), education (less than high school, high school graduate, some college, college graduate and beyond), region of the country, (West, Northeast, Midwest, South), and tobacco use (former or current smoker vs. never). Models stratified by sex were adjusted for race; models stratified by race were adjusted for sex.

## Supplementary Materials

The following are available online at http://www.mdpi.com/1660-4601/15/9/1965/s1, Table S1: Prevalence of lifetime allergies by mental health outcomes.
