# Supplementary material for: Seasonal Allergies and Psychiatric Disorders in the United States
Source: Int J Environ Res Public Health. 2018 Sep 8;15(9):1965. doi: 10.3390/ijerph15091965 (PMC6164754; doi:10.3390/ijerph15091965)
Supplement: Supplementary file 1 [file ijerph-15-01965-s001.pdf]

Table S1. Prevalence of lifetime allergies by mental health outcomes

	Lifetime Allergies % (SE)	95% CI	F-statistic (p-value)
Mood Disorders			
Yes	43.75 (1.24)	0.41-0.46	29.44 (p<0.00)
No	34.87 (1.32)	0.32-0.37	
Anxiety Disorders			
Yes	43.53 (1.08)	0.41-0.46	60.44 (p<0.00)
No	32.58 (1.37)	0.30-0.35	
Substance Use Disorders			
Yes	38.59 (2.52)	0.34-0.44	0.50 (0.48)
No	36.42 (1.28)	0.34-0.39	
Alcohol Use Disorders			
Yes	38.15 (2.00)	0.34-0.42	0.60 (0.44)
No	36.35 (1.28)	0.34-0.39	
Eating Disorders			
Yes	45.67 (3.41)	0.39-0.52	7.89 (0.01)
No	36.38 (1.16)	0.34-0.39	
